# Effect of Water Extract of *Artemisia annua* L. on Growth Performance, Blood Biochemical Parameters and Intestinal-Related Indices in Mutton Sheep

**DOI:** 10.3390/ani16020340

**Published:** 2026-01-22

**Authors:** Gen Gang, Ruiheng Gao, Manman Tong, Shangxiong Zhang, Shiwei Guo, Xiao Jin, Yuanyuan Xing, Sumei Yan, Yuanqing Xu, Binlin Shi

**Affiliations:** 1 College of Animal Science, Inner Mongolia Agricultural University, Hohhot 010018, China; ganggen@emails.imau.edu.cn (G.G.); gaoruiheng@emails.imau.edu.cn (R.G.); nndtmm@163.com (M.T.); guoshiwei@emails.imau.edu.cn (S.G.); yaojinxiao@aliyun.com (X.J.); xingyuanyuan2014@163.com (Y.X.); yansmimau@163.com (S.Y.); 2 National Center of Pratacultural Technology Innovation (Under Preparation), Hohhot 010060, China; zhangshangx@126.com

**Keywords:** growth performance, blood biochemical index, intestinal-related index, mutton sheep, water extracts of *Artemisia annua* L.

## Abstract

Objective: This experiment aimed to explore the effects of water extract of *Artemisia annua* L. (WEAA) on growth performance, blood parameters, and intestinal-related indices in mutton sheep, so as to evaluate its potential as a natural growth promoter. Methods: The experiment was conducted using a completely randomized design. Thirty-two 3-month-old Dorper × Han mutton sheep were randomly assigned to 4 groups (n = 8). The control group was fed only the basal diet, while the other groups were fed the basal diet supplemented with, respectively, 500, 1000, and 1500 mg/kg WEAA. The adaptation period lasted 15 days, followed by a 60-day experimental period. Results: Results showed that dietary supplementation of WEAA significantly reduced average daily feed intake (ADFI) and feed-to-gain ratio (F:G) of mutton sheep, significantly improved the apparent digestibility of crude protein (CP) and phosphorus (P), and optimized blood biochemical indices, such as significantly increasing the concentrations of total protein (TP), albumin (ALB), high-density lipoprotein cholesterol (HDL-C), and glucose (GLU), while significantly decreasing blood urea nitrogen (BUN) level (*p* < 0.05). Additionally, WEAA significantly improved intestinal morphology by reducing the crypt depth (CD) of the duodenum, jejunum, and ileum, increasing jejunal villus height (VH), and elevating the villus-to-crypt ratio (VH/CD) across intestinal segments (*p* < 0.05). It also significantly enhanced the activity of intestinal digestive enzymes, including α-amylase and trypsin in the duodenum, lipase and chymotrypsin in the jejunum, and α-amylase and chymotrypsin in the ileum, with the 500 mg/kg and 1000 mg/kg WEAA groups reaching better activity (*p* < 0.05). Furthermore, WEAA supplementation significantly increased the counts of beneficial bacteria (*Bifidobacteria* and *Lactobacilli*) and decreased the count of harmful bacteria (*Escherichia coli*) in rectal fecal samples (*p* < 0.05). Notably, most of these beneficial effects were dosage-dependent, with overall optimal performance observed in the 1000 mg/kg WEAA group. Conclusion: In conclusion, supplementing the diet with 1000 mg/kg WEAA exerted significant positive effects on the feed efficiency, nutrient digestibility, and intestinal health status of mutton sheep.

## Simple Summary

Various plant extracts are used as functional nutritional factors to improve animal health and production performance. This experiment investigated the effects of water extract of *Artemisia annua* L. (WEAA) on growth performance, biochemical parameters, and intestinal-related indicators in mutton sheep. The results showed that dietary supplementation with WEAA could significantly improve nutrient digestibility and intestinal health without affecting growth performance, which provides technical support for efficient and healthy breeding of mutton sheep and exhibits certain practical application value.

## 1. Introduction

The global demand for high-quality mutton is steadily increasing, driving the mutton sheep breeding industry towards greater intensification and efficiency. In this context, enhancing growth performance, improving feed utilization, and ensuring product quality have become paramount objectives for sheep farming [1,2]. However, intensive production systems often present significant challenges, including metabolic stress, a lower feed conversion rate, and a higher incidence of intestinal health disorders [3]. Intestinal health, in particular, is a critical determinant of overall animal well-being and productivity, as it is related to nutrient digestion and absorption. Issues such as imbalanced gut microbiota and compromised intestinal barrier function can severely restrict growth performance and economic returns, while also potentially impacting the safety and quality of the final mutton products [4]. For decades, antibiotic growth promoters were widely used to mitigate these issues and enhance performance. However, growing concerns over antibiotic residues in animal products and the emergence of antimicrobial resistance have led to their ban or restriction in many countries [5]. This has spurred a global search for green, safe, and effective alternatives. Natural plant extracts and their bioactive compounds have emerged as promising candidates, offering a multi-faceted promoting effect on animal health and nutrition. These phytogenic additives are valued for their lack of residues, low risk of resistance development, and their combined nutritional and health-promoting properties [6]. Consequently, identifying and validating natural extracts that can effectively enhance production performance and regulate intestinal health in mutton sheep is of significant practical importance for advancing the sustainable development of the industry.

*Artemisia annua* L., also known as Qinghao, is a medicinal herb with a long history of use in traditional Asian medicine [7]. It is recognized as a rich source of various bioactive substances and is considered a potential natural feed additive [8]. The biological activity of this plant is attributed to its diverse array of secondary metabolites, including the well-characterized sesquiterpene lactone artemisinin, along with flavonoids, phenolic acids, coumarins, and essential oils [9,10]. The water extract of *Artemisia annua* L. (WEAA) contains a complex mixture of these compounds, which may act synergistically to exert various biological effects [11]. Notably, water extraction was selected as the extraction method for this study because it can effectively retain active water-soluble components such as polysaccharides and flavonoids. This method offers greater cost-effectiveness and environmental safety, avoids solvent residue issues, and is more suitable for large-scale application in animal production. Existing studies have highlighted the biological functions of *Artemisia annua* L. extract in animal production, such as promoting growth performance [12], antioxidation [13,14,15], anti-inflammation [10,16], antimicrobial and antiviral activities [17] and regulating the intestinal flora [8]. Among these, studies on broilers have demonstrated that *Artemisia annua* L. polysaccharides can increase the jejunal villus-to-crypt ratio (VH/CD), enhance the structural integrity of the intestinal mucosa by improving mucosal morphological parameters, and thereby provide structural support for nutrient absorption and intestinal barrier function [18]. More directly, Guo et al. (2023) [8] reported that dietary supplementation with water extract of *Artemisia annua* L. (WEAA) significantly improved broilers’ final body weight and feed efficiency, reduced feed intake, increased digestive enzyme activity, decreased intestinal crypt depth (CD), promoted the proliferation of beneficial *Lactobacilli*, and inhibited the growth of pathogenic *Escherichia coli*. Similar beneficial effects have been documented in other monogastric animal species. In the experiment of Eimeria-infected growing rabbits, Abousekken et al. (2015) [12] found that dietary supplementation with *Artemisia annua* L. powder improved average daily gain (ADG), optimized feed-to-gain ratio (F:G), and decreased the total bacterial count in the cecum. In addition, Niu et al. (2020) [19] demonstrated that adding 2 g/kg of enzymatically treated *Artemisia annua* L. extract (rich in flavonoids and polyphenols) to the diet optimally enhanced the growth performance and intestinal health of weaned piglets by strengthening antioxidant capacity and alleviating oxidative damage. Furthermore, Xiong et al. (2023) [20] confirmed that supplementation with 1.0 g/kg of an enzymatically treated *Artemisia annua* L. extract during late gestation and lactation increased the average daily feed intake (ADFI) of heat-stressed sows and the ADG of their preweaning offspring; simultaneously, it increased the villus height of the duodenum and ileum in piglets, thereby promoting intestinal development. Despite this promising foundation, research on the application of WEAA in ruminants remains limited. Previous studies have shown that WEAA can significantly enhance the immune performance of sheep, strengthen their antioxidant capacity, and also have a positive effect on the balance and stability of the rumen microbial flora [18]. However, the application effect of WEAA in the sheep feed has not been systematically studied so far. There is a significant gap in the research on the impact of WEAA on nutrient digestibility, intestinal morphology, digestive enzyme activity and intestinal flora of mutton sheep.

Based on the previous research results, it was hypothesized that dietary supplementation with WEAA would improve the growth performance of mutton sheep by increasing apparent nutrient digestibility and regulating blood biochemical parameters related to nutrient metabolism and immunity. Furthermore, it was postulated that WEAA would improve intestinal-related indices, thereby maintaining intestinal health. So, this study focused on the application of WEAA in mutton sheep, systematically explore the comprehensive effects of WEAA on growth performance, nutrient utilization, and intestinal health, while clarifying the dose-effect relationship of WEAA in sheep diets, in order to provide a scientific basis for formulating precise feeding strategies.

## 2. Materials and Methods

### 2.1. Animals, Treatments, and Experimental Conditions

*Artemisia annua* L. was harvested in Hohhot, Inner Mongolia. The plant material was shade-dried, cut into segments, and subjected to water extraction. The extract was concentrated and freeze-dried to obtain a powder, which was stored at 4 °C until use. The detailed production procedures were performed as described previously [21], and all samples were maintained at a temperature of 4 °C to ensure stability, and the chemical ingredients of WEAA are listed in Supplementary Table S1, Figures S1 and S2.

The animals used in this study were purchased from a livestock farm in Ordos City, Inner Mongolia Autonomous Region, China. The animal experiment protocol was approved by the Animal Care and Use Committee of Inner Mongolia Agricultural University (approval code: NND2021097; approval date: 5 November 2021).

The experiment was carried out from September to December 2022 at the experimental station of Inner Mongolia Agricultural University in Hohhot, Inner Mongolia. Prior to the experiment, the sheep were allowed a 7-day acclimation period to recover from transport stress and adapt to the new environment. During this period, health observations were conducted daily, including monitoring of feeding behavior and mental status, to ensure the sheep were healthy and fit for the trial. Deworming was also performed to optimize gastrointestinal function. The experiment was conducted using a completely randomized design. Thirty-two healthy mutton sheep (three-month-old female sheep, Dorper × Han) with 24.00 ± 2.51 kg (mean ± SD) body weight were allotted into four treatment groups (n = 8). The control group received only basal diet; experimental groups were fed the same basal diet supplemented with 500, 1000, and 1500 mg/kg of diet, respectively, based on prior studies reporting effective doses of *Artemisia* extracts in ruminant nutrition [22,23,24]. All sheep were housed individually in stalls (2.4 m × 3.6 m) within a naturally ventilated barn and maintained under consistent management conditions throughout the experiment. The adaptation period lasted 15 days, followed by a 60-day experimental period. Each sheep in the trial received the pelleted rations twice a day at 8:00 a.m. and 16:00 p.m. WEAA is added to the basal diet in proportion during the process of diet formulation. The amount of feed offered was adjusted daily to maintain a refusal rate not less than 5%, ensuring ad libitum access to feed and water. Daily health monitoring was performed to detect any abnormalities promptly, thereby ensuring the integrity of experimental data and animal welfare. Basal diet ingredients and nutrient profiles are presented in Table 1.

### 2.2. Growth Performance and Nutrient Apparent Digestibility

Body weights were measured in the fasting state at 07:00 on d 0, 30, and 60 of the trial period, respectively. ADG was calculated as the total weight gain per sheep divided by the number of days in each corresponding period. During the trial, the daily amounts of feed and feed refusals were recorded every morning to determine the ADFI. The F:G was calculated based on ADG and ADFI.

Feed and fecal samples were collected, weighed, and mixed systematically over 7 consecutive days, specifically from d 24 to 30 and from d 54 to 60 of the trial period. Feed and fecal samples were first oven-dried at 65 °C for 72 h until constant weight, then ground to pass through a 1 mm screen [25]. Dry matter (DM) content was subsequently determined by oven drying (GB/T 6435-2014) at 105 °C for 8 h using a DGX-9053B drying oven (Fuma Experimental Equipment Co., Ltd., Shanghai, China). Crude protein (CP) was quantified via the Kjeldahl method (GB/T 6432-2018) using a K9840 auto-analyzer (Haineng Scientific Instrument Co., Ltd., Dezhou, Shandong, China). Ether extract (EE) was determined by Soxhlet extraction (GB/T 6433-2006) using a SOX406 analyzer (Haineng Scientific Instrument Co., Ltd., Shandong, China). The ash content was determined by combustion in a muffle furnace at 550 °C for 3 h (GB/T 6438-2007). Neutral detergent fiber (NDF) and acid detergent fiber (ADF) were analyzed using the filter bag method (GB/T 20806-2006 and NY/T 1459-2007), with an ANKOM A200i Fiber Analyzer (ANKOM Technology, New York, NY, USA). Calcium (Ca) in the samples was determined by the potassium permanganate method (GB/T 6436-2018), and phosphorus (P) was analyzed via the spectrophotometric method (GB/T 6437-2018) using a UV-visible spectrophotometer (Model: 752S, Lengguang Tech., Shanghai, China), respectively [26,27,28,29,30,31,32,33].

### 2.3. Preparation and Analysis of Blood Samples

Samples were collected from each sheep on d 30 and 60 of the trial, prior to the morning feeding. After collection, the blood samples were centrifuged at 3500 rpm for 15 min to separate the serum, which was then stored at −20 °C until analysis. Blood biochemical parameters, including total protein (TP), albumin (ALB), blood urea nitrogen (BUN), glucose (GLU), triglyceride (TG), total cholesterol (TC), high-density lipoprotein cholesterol (HDL-C), low-density lipoprotein cholesterol (LDL-C), creatinine (CRE), alkaline phosphatase (ALP), total bilirubin (TBIL), were measured using commercial kits purchased from Nanjing Jiancheng Bioengineering Institute (Nanjing, China). All the analyses were performed according to the manufacturer’s instructions.

### 2.4. Measurement of Intestinal Morphology and Enzymatic Activity

On d 60 of the trial, all 32 sheep (4 groups × 8 replicates, 1 lamb per replicate) were fasted for 12 h and then slaughtered, exsanguinated via the jugular vein to induce death, according to the animal welfare and euthanasia rules described by the National Standard Operating Procedures (GB/T 43562-2023, China) [34]. Then, the tissue samples from the duodenum, jejunum, and ileum were collected and immediately immersed in 10% neutral-buffered formalin (fixative volume ≥ 10 × tissue volume) and stored at 4 °C. Fixed tissues were paraffin-embedded and sectioned into 5-μm slices for hematoxylin and eosin (H&E) staining. Subsequently, intestinal VH and CD were measured using an image analysis system at 200× magnification. The VH/CD ratio was calculated as the mean villus height divided by the mean crypt depth. Then, chyme samples were collected from each intestinal segment and stored at −20 °C. The sample was used for α-amylase, lipase, trypsin, and chymotrypsin activity measurement with commercial kits (Nanjing Jiancheng Bioengineering Institute, Nanjing, China) according to the manufacturer’s protocols.

### 2.5. Bacterial Enumeration of Intestinal Flora

Rectal fecal samples were collected using sterile gloves from sheep at 8:00 a.m. on d 30 and 60 of the experimental period, prior to feeding. Samples were immediately stored at −80 °C for subsequent microbiota analysis, as described below [35].

Rectal fecal samples were collected and immediately snap-frozen, then transported to the laboratory in a liquid nitrogen tank and stored at −80 °C to maintain bacterial viability until analysis. The frozen samples were thawed rapidly in a 37 °C water bath for 10 min before processing, then each 0.5 g rectal content sample was accurately weighed and mixed with 4.5 mL sterile physiological saline, vortexed thoroughly for 30 s to obtain a homogeneous 10^−1^ dilution suspension, followed by serial 10-fold dilutions to generate seven gradients (10^−1^ to 10^−7^) with each step vortexed for 30 s to ensure uniform mixing. For aerobic culture, 0.3 mL of the appropriate dilution (selected based on preliminary experiments) was spread onto nutrient agar (Cat. No. 022020, for total aerobic bacteria) and eosin methylene blue (EMB) agar (Cat. No. 022060, for *Escherichia coli*, respectively, with 3 replicates per gradient, and the inoculated Petri dishes were incubated aerobically at 37 °C for 24 h; for anaerobic culture, 0.15 mL of the appropriate dilution was injected into anaerobic tubes containing nutrient agar (Cat. No. 022020, for total anaerobic bacteria), BBL™ (Becton, Dickinson and Company, Franklin Lakes, NJ, USA) agar (Cat. No. 027330, for *Bifidobacteria*), and MRS (de Man, Rogosa and Sharpe) broth medium (Cat. No. 027312, for *Lactobacilli*, respectively, with 3 replicates per gradient. All commercial selective media were purchased from Guangdong Huankai Microbial Science and Technology Co., Ltd. (Guangzhou, China), and the anaerobic tubes were incubated anaerobically at 37 °C for 48 h under an anaerobic atmosphere (5% CO_2_ balanced with N_2_). After incubation, Petri dishes and anaerobic tubes with 50~150 colonies were selected for counting, two suitable consecutive gradients were used to calculate the average number of colonies, colony-forming unit (CFU)/g was computed using the formula: CFU/g = (Average number of colonies × Dilution factor)/Volume of inoculum (mL), and all results were expressed as logarithmic values (Log CFU/g).

### 2.6. Statistical Analysis

Raw data were entered and organized using Microsoft Excel 2016. All statistical analyses were performed using SAS Version 9.2 (SAS Institute Inc., Cary, NC, USA). The PROC UNIVARIATE procedure was utilized to screen the data for normality and identify outliers. Any obvious outliers or missing values identified were verified against the original experimental records and excluded from the final analysis to ensure data quality. Data were analyzed using the PROC GLM procedure for one-way analysis of variance (ANOVA) to test for overall differences among treatments. Differences among treatment means were separated using Duncan’s multiple range test. Orthogonal polynomial contrasts were performed to determine the linear and quadratic effects of increasing dietary WEAA levels on the measured indices. Results were reported as mean and standard error of the mean (SEM), with *p* < 0.05 as the threshold for statistical significance.

## 3. Results

### 3.1. Growth Performance

**Figure 1:**
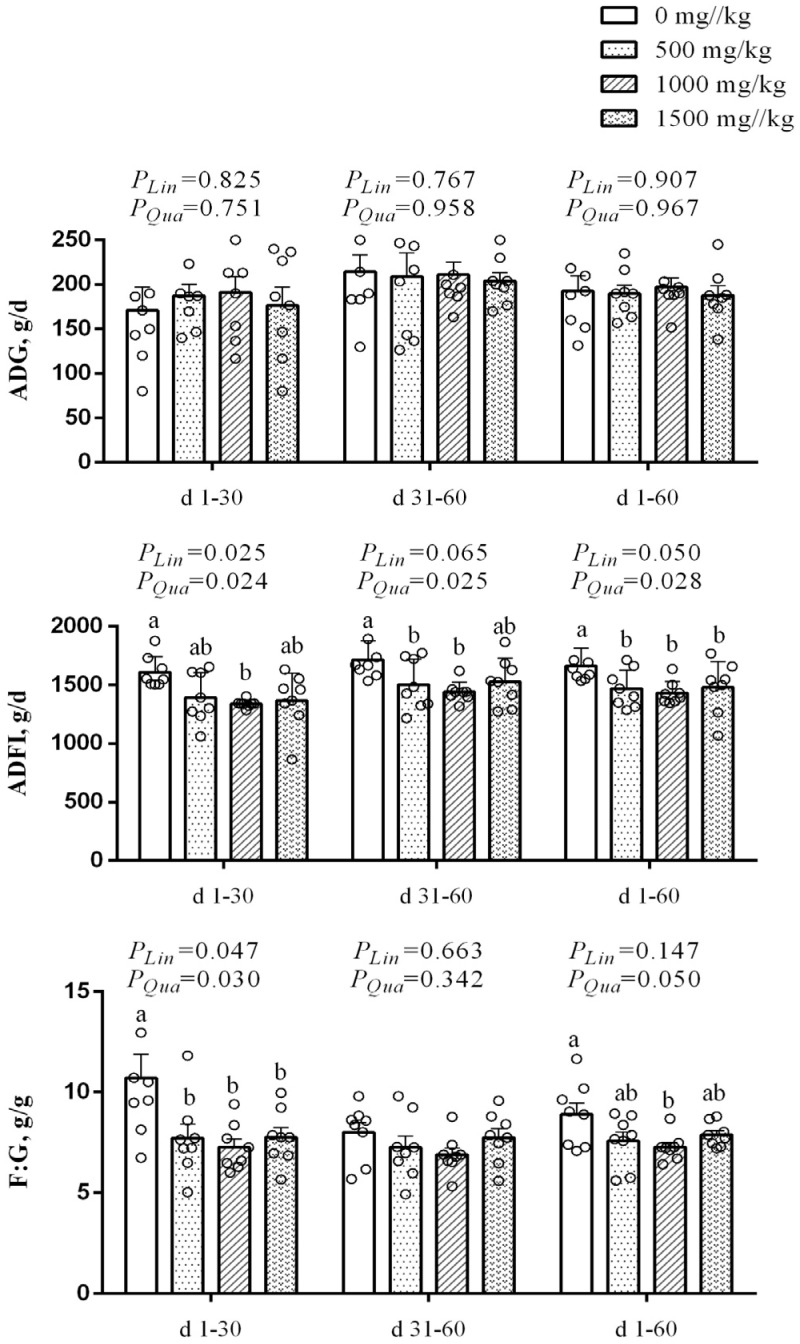
Growth performance of mutton sheep fed diets supplemented with different levels of WEAA. WEAA: Water extracts of *Artemisia annua* L.; Dose-dependent effects of WEAA (0, 500, 1000, 1500 mg/mL), values were expressed as the means of 8 sheep in each group. Dose-dependent effects of WEAA (Lin: Linear; Qua: Quadratic); ^a,b^ Different letters indicate significant differences between mean values for a given indicator (*p* < 0.05), whereas the probability value of 0.05 ≤ *p* < 0.10 was considered as a tendency (±standard error).

Figure 1 showed the effect of dietary WEAA on the growth performance in mutton sheep. From d 1 to 30, with increasing dietary WEAA supplementation, ADFI exhibited a significant linear or quadratic decrease (*p* < 0.05), the 1000 mg/kg WEAA supplementation significantly reduced ADFI compared to the control group (*p* < 0.05). From d 31 to 60, a significant quadratic decrease (*p* < 0.05) was observed, with WEAA at 500 and 1000 mg/kg significantly lowering ADFI relative to the control (*p* < 0.05). Over the entire period (d 1 to 60), ADFI showed a linear decrease (*p* = 0.050) and a significant quadratic decline (*p* < 0.05), and WEAA supplementation reduced ADFI compared to the control (*p* = 0.05). From d 1 to 30, with increasing dietary WEAA supplementation, F:G exhibited a significant linear or quadratic decrease (*p* < 0.05), and WEAA supplementation significantly reduced F:G compared to the control group (*p* < 0.05).

### 3.2. Nutrient Apparent Digestibility

As depicted in Table 2, different dietary WEAA supplementation levels influenced the apparent digestibility of nutrients in mutton sheep. On d 60, the apparent digestibility of CP presented a significant quadratic increase (*p* < 0.05), and the apparent digestibility of P showed a significant linear or quadratic increase (*p* < 0.05), with the 500 and 1000 mg/kg WEAA groups significantly higher than the control (*p* < 0.05). There was no significant difference in the apparent digestibility of DM, NDF, ADF, Ca between the WEAA-supplemented groups and the control group (*p* > 0.05).

### 3.3. Blood Biochemical Indicators

Table 3 showed the effects of dietary WEAA supplementation at different levels on the blood biochemical indicators of mutton sheep. On d 30, with increasing dietary WEAA supplementation, the TP and ALB concentration exhibited a linear or quadratic increase (*p* < 0.05), and the 1000 and 1500 mg/kg WEAA groups were significantly higher than the control group (*p* < 0.05). HDL-C concentrations exhibited a significant quadratic increase (*p* < 0.05), and the 1000 mg/kg WEAA group was significantly higher than the control group (*p* < 0.05). On d 60, with increasing dietary WEAA supplementation, the concentration of TP, ALB and GLU exhibited a linear or quadratic increase (*p* < 0.05), and the 500 and 1000 mg/kg WEAA groups were significantly higher than the control group (*p* < 0.05). The BUN concentration exhibited a linear or quadratic decrease (*p* < 0.05), and the 1000 mg/kg WEAA group was significantly lower than the control group (*p* < 0.05).

### 3.4. Intestinal Morphology

The effects of dietary WEAA supplementation on intestinal morphology of mutton sheep are presented in Table 4. Duodenal CD significantly decreased in a linear or quadratic manner with increasing WEAA supplementation (*p* < 0.05), VH/CD ratio showed a linear increase (*p* < 0.05), and duodenal CD was lower in the 1000 mg/kg WEAA group compared to the control (*p* < 0.05); jejunal VH showed a significant linear increase (*p* < 0.05), CD exhibited a significant linear or quadratic increase (*p* < 0.05), and jejunal VH/CD ratio significantly increased in a linear or quadratic manner with increasing WEAA supplementation (*p* < 0.05), and jejunal CD was lower in the WEAA groups, the VH/CD ratio was higher in the 1000 and 1500 mg/kg WEAA groups compared to the control (*p* < 0.05); ileal CD exhibited a linear or quadratic decrease (*p* < 0.05), and the 1000 mg/kg WEAA group significantly lower than the control (*p* < 0.05), while ileal VH/CD ratio showed a significant linear or quadratic increase (*p* < 0.05) with increasing WEAA supplementation, and the 1000–1500 mg/kg WEAA groups were significantly higher than the control (*p* < 0.05).

### 3.5. Intestinal Digestive Enzymes

Table 5 shows the effect of dietary WEAA supplementation on the activity of intestinal digestive enzymes in mutton sheep. With increasing WEAA levels, duodenal α-amylase activity exhibited a significant linear or quadratic increase (*p* < 0.01), while ileal α-amylase showed a quadratic increase (*p* < 0.05), and the 1000 mg/kg WEAA group was significantly higher than the control (*p* < 0.05). Jejunum lipase activity significantly increased in a quadratic manner (*p* < 0.05), chymotrypsin activity in the jejunum and ileum presented a significant linear or quadratic increase (*p* < 0.05), and all WEAA groups were significantly higher than the control (*p* < 0.05). Trypsin activity in the duodenum exhibited a linear increase (*p* < 0.05).

### 3.6. Bacterial Enumeration of Intestinal Flora

As shown in Table 6, dietary WEAA supplementation affected the bacterial enumeration of intestinal flora in mutton sheep. On d 30, no significant linear or quadratic changes were observed in the counts of total aerobic bacteria, *Escherichia coli*, total anaerobic bacteria, *Bifidobacteria* or *Lactobacilli* with increasing WEAA levels (*p* > 0.05). On d 60, *Escherichia coli* counts exhibited a quadratic decrease (*p* < 0.05), and the 1000 mg/kg WEAA group was significantly lower than the control (*p* < 0.05). *Bifidobacteria* counts showed a significant linear or quadratic increase (*p* < 0.05), and the 1000 mg/kg WEAA group was significantly higher than the control (*p* < 0.05). *Lactobacilli* significantly increased in a quadratic manner (*p* < 0.05), and the 500 and 1000 mg/kg WEAA groups were significantly higher than the control (*p* < 0.05).

## 4. Discussion

### 4.1. Growth Performance and Nutrient Apparent Digestibility

*Artemisia annua* L. is a traditional Chinese medicinal plant rich in bioactive components including polysaccharides, flavonoids, and sesquiterpenes [7,13]. Decoction is a common processing method for this plant, which facilitates the breakdown of cell walls and the subsequent release of bioactive ingredients to exert pharmacological effects [8,17,36,37]. The enhancement of nutrient apparent digestibility represents a core mechanism by which plant bioactive contents enhance feed efficiency and growth performance in ruminants. In this study, although dietary supplementation with WEAA did not significantly alter ADG in mutton sheep, it significantly reduced ADFI and F:G, and significantly improved the apparent digestibility of CP and P. These findings are consistent with previous studies demonstrating that *Artemisia*-derived extracts can optimize growth performance and feed utilization efficiency in animals [38,39,40,41]. For instance, *Artemisia annua* L. extract has been shown to reduce F:G, improve feed conversion efficiency in sheep [41]. In the feeding experiments of weaned piglets and lambs, extracts of *Artemisia* plants were found to significantly decrease ADFI without significant changes in ADG [40,41]. Optimal animal production performance is tightly linked to nutrient digestion and metabolic efficiency [42]. Enhancing nutrient digestibility promotes the absorption and biotransformation of key nutrients, thereby exerting a positive regulatory effect on growth performance [39]. Previous research has confirmed that plant extracts, including those from *Artemisia* species, can improve the apparent digestibility of CP in sheep [16,43], which aligns with the elevated CP digestibility observed in the present study. This improvement often exhibits a dose-dependent, quadratic pattern, with beneficial effects observed at moderate supplementation levels, as also reported for *Salvia sclarea* L. extract supplementation has been shown to enhance CP and NDF digestibility in sheep [44], further supporting the viewpoint that plant-derived bioactive compounds contribute to improve nutrient utilization. The efficacy of WEAA in optimizing feed efficiency and nutrient digestibility is presumably attributed to its abundant bioactive constituents, including organic acids, polysaccharides, flavonoids, and terpenoids. These compounds collectively promote nutrient digestion and absorption, with specific enhancements in the digestibility and utilization of CP and P. This allows mutton sheep to acquire sufficient nutrients for growth despite reduced ADFI, thereby maintaining stable ADG while decreasing F:G and improving overall feed utilization efficiency.

### 4.2. Effects on Blood Biochemical Parameters

Blood biochemical parameters serve as core biological markers for evaluating metabolic status, nutrient utilization efficiency, and organ function, with their specific alterations precisely reflecting the targeted regulatory effects of functional nutrients on animal physiological metabolism [39,45]. The present study focused on key indicators of protein metabolism, energy metabolism, and lipid metabolism, as well as markers of hepatic and renal function, to systematically assess the effects of WEAA on metabolic processes in mutton sheep. The results demonstrated that WEAA significantly increased the levels of serum ALB, HDL-C, GLU, and TP, while significantly reducing BUN. These changes indicate that the metabolic regulation of WEAA in mutton sheep primarily focuses on optimizing protein synthesis, enhancing nitrogen metabolism efficiency, and improving energy supply, with no significant regulatory effects on hepatic and renal function or overall lipid metabolism. These findings are in line with previous studies showing that plant-derived extracts can modulate blood biochemical parameters related to protein and energy metabolism in animals. For example, *Artemisia annua* L. extract supplementation significantly elevated serum ALB levels and optimized hematological parameters in rainbow trout [45], which attributed to its rich content of polysaccharides, flavonoids, terpenoids, and phenolic compounds [46]. Similarly, dietary inclusion of multi-herbal additive increased serum ALB concentration while decreasing serum urea levels in lambs [47]. Such consistency suggests a shared mechanism by which plant bioactive components regulate nitrogen and protein metabolism across different animal species. Mechanistically, the active components in WEAA may enhance digestive enzyme activity, promote the digestion and absorption of proteins and carbohydrates, and alleviate nutritional antagonism, thereby providing adequate substrates for hepatic protein synthesis. This regulatory pathway corresponds with the enhanced CP apparent digestibility observed in the present study, ultimately leading to significantly elevated serum ALB and TP levels. The significant reduction in BUN likely stems from more efficient absorption and utilization of dietary nitrogen by mutton sheep, reducing the excretion of unutilized nitrogen, which was highly consistent with the optimized F:G ratio in production performance results. Notably, the significant elevation of serum GLU further supports the role of WEAA in improving energy metabolism in mutton sheep, potentially through promoting intestinal carbohydrate absorption or optimizing hepatic glycogen metabolism to provide a stable energy supply for growth and development. A limitation of this study is that intermediate metabolites of amino acid metabolism (such as branched-chain amino acids and glutamine) were not measured. Future research may integrate metabolomics technologies to further elucidate the molecular mechanisms for WEAA’s regulation action on the metabolism in sheep.

### 4.3. Intestinal-Related Indicators

The villus-crypt structure of the intestinal mucosa serves as the structural foundation for nutrient absorption surface area and barrier function, with changes in its morphological parameters (CD, VH/CD) directly affecting nutrient absorption efficiency and intestinal health [8]. In the present study, WEAA significantly reduced CD in the duodenum, jejunum, and ileum of mutton sheep, while significantly increasing VH/CD. This morphological optimization provided important structural support for the efficient absorption of nutrients, particularly CP and P. Previous studies have demonstrated that plant extracts can improve intestinal mucosal morphology in various animal species. For example, *Artemisia* polysaccharides relieved lipopolysaccharide-induced crypt damage and restored normal intestinal cell proliferation in mice [48]. Although the reduction in CD by WEAA in the present study differs from the inflammatory model, both reflect the positive regulatory effects of plant-derived substances on intestinal morphology. Based on the experimental results, it is speculated that WEAA may inhibit excessive crypt cell proliferation, reduce ineffective metabolic consumption, and enhance intestinal barrier function to minimize nutrient loss and harmful substance invasion. This optimization of intestinal morphology establishes an important structural foundation for improving the digestibility of nutrients such as CP and P.

Optimization of intestinal morphology not only furnishes a structural foundation for nutrient absorption but also enhances the secretory capacity of intestinal mucosal epithelial cells, thereby modulating the synthesis and activity of digestive enzymes [19]. Research indicates that plant-derived feed additives, rich in nutritional elements and bioactive components, can activate gastrointestinal function in animals, promote digestive gland secretion of digestive enzymes, and accelerate the digestion and absorption of nutrients in feed [49,50], thus improving feed utilization efficiency and nutrient absorption capacity. The synthesis and activity regulation of intestinal digestive enzymes represent key steps in converting dietary nutrients into absorbable small molecules, and their efficient function directly depends on the dual support of amino acids as synthetic substrates and regulatory signals [8,49]. In the present study, WEAA significantly enhanced amylase activity in the duodenum and ileum, lipase activity in the jejunum, chymotrypsin activity in the jejunum and ileum, and trypsin activity in the duodenum, revealing the regulatory value of WEAA on nutrient digestion in mutton sheep from the perspective of enzymatic reaction substrates. These results align with previous studies showing that plant polysaccharides can enhance intestinal digestive enzyme activity in animals. For instance, spirulina powder supplementation increased intestinal α-amylase, lipase, and trypsin activity in growing quails [51], and *Astragalus* polysaccharides enhanced amylase, lipase, and protease activity in broiler intestinal segments [52]. Mechanistically, WEAA supplementation may provide sufficient and balanced amino acid substrates for the synthesis of α-amylase, lipase, and protease, ensuring the efficiency of enzyme protein synthesis. Simultaneously, the optimization of intestinal morphology (increased VH/CD) improves the secretory function of intestinal mucosal epithelial cells, creating a favorable microenvironment for the release and action of digestive enzymes. Notably, the increased trypsin and amylase activity in the duodenum strengthen the preliminary breakdown of nutrients, the enhanced lipase activity in the jejunum specifically improves fat digestion efficiency, and the increased chymotrypsin and amylase activity in the ileum promote thorough digestion of incompletely decomposed nutrients. The quadratic responses in digestive enzyme activities suggest that moderate WEAA levels optimally enhance enzymatic function, while higher doses may exert inhibitory effects, thereby reducing digestive capacity and potentially disrupting microbial balance.

Enhanced digestive enzyme activity facilitates the efficient decomposition of nutrients, provides appropriate substrates for the growth and metabolism of intestinal flora, and thereby modulates the structural balance of the intestinal microbiota [8,39]. The interaction between intestinal microbiota and host is dynamic equilibrium of nutrient competition-metabolic synergy, and the changes in the ratio of beneficial bacteria (*Bifidobacteria*, *Lactobacilli*) to harmful bacteria (*Escherichia coli*) are directly associated with intestinal nutrient conversion efficiency and mucosal barrier stability [18,48]. In this study, dietary supplementation with WEAA reduced the count of *Escherichia coli* in the intestines of mutton sheep, while significantly increasing the counts of *Bifidobacteria* and *Lactobacilli* (beneficial bacteria), and this change forms a synergistic effect with intestinal morphological optimization and enhanced digestive enzyme activity. Abdel-Wahab et al. (2023) [51] also found that dietary supplementation with Spirulina platensis powder in quails significantly increased the abundance of *Lactobacilli* in the intestine, while significantly inhibiting *Escherichia coli* and *Salmonella*. The results of the present experiment further confirm this regulatory pattern: dietary supplementation with WEAA significantly reduced the number of *Escherichia coli* in rectal fecal samples of mutton sheep, while increasing the numbers of *Lactobacilli* and *Bifidobacteria*, indicating that it could improve intestinal health and support growth performance enhancement through optimizing intestinal microbiota structure. It is noteworthy that previous research has found that dietary supplementation with WEAA in sheep can significantly increase ruminal *Bifidobacteria* abundance. Combined with the results of this experiment, the regulatory mechanism may involve two aspects: firstly, WEAA can serve as a fermentation substrate for *Bifidobacteria* and *Lactobacilli*, directly providing energy and nutrition for the proliferation of beneficial bacteria, promoting their colonization advantage in the intestine; secondly, the enhanced intestinal mucosal barrier integrity observed in earlier studies (reduced CD, increased VH/CD) can reduce the adhesion and colonization of *Escherichia coli* on the intestinal epithelium, while acetic acid and propionic acid produced by the proliferation of beneficial bacteria can lower intestinal pH, further inhibiting the growth and reproduction of harmful bacteria. The synergistic effect of intestinal barrier enhancement and microbiota balance not only sustains intestinal health but also diminishes the production of harmful bacterial metabolites, alleviates the body’s metabolic burden, and facilitates the efficient absorption of nutrients, contributing to the reduced F:G and optimized growth performance of mutton sheep. In practical application, the use of WEAA as a feed additive for mutton sheep also exhibits certain economic advantages. *Artemisia annua* L. is widely available and easy to process, and the water extraction method is relatively low-cost. The supplementation level of 1000 mg/kg WEAA was considered relatively better in this study. Collectively, these characteristics suggest that WEAA may provide a cost-effective option for improving the productivity of mutton sheep under practical feeding conditions. Future research should focus on identifying the specific active components of WEAA and verifying its long-term effects under commercial production conditions.

## 5. Conclusions

This study demonstrated that dietary supplementation of WEAA reduced the ADFI and F:G of mutton sheep, enhanced the apparent digestibility of EE, CP, and P, and modulated blood biochemical profiles—specifically elevating the concentration of TP, ALB, HDL-C, and GLU, while reducing BUN levels. Additionally, WEAA improved intestinal histological morphology and enhanced digestive enzyme activity in specific intestinal segments. Furthermore, WEAA modulated gut microbiota homeostasis by suppressing the proliferation of *Escherichia coli* and promoting the growth of *Bifidobacteria* and *Lactobacilli*. Notably, the effects of WEAA supplementation showed a dose-dependent pattern, with the 1000 mg/kg group exhibiting relatively better comprehensive efficacy. Overall, dietary WEAA supplementation synergistically improved feed efficiency, nutrient digestibility, and intestinal health in mutton sheep. This provides technical support for the efficient and healthy breeding of mutton sheep, with considerable practical application value.

## Figures and Tables

**Table 1:** Ingredients and nutrient levels of experimental diets (%, dry matter (DM) basis).

Item	Content
Ingredient composition	
Alfalfa	15.95
Corn straw grass	13.75
Oat grass	25.08
Corn	23.32
Soybean meal	10.71
Wheat bran	4.30
Corn germ meal	1.86
Soybean oil	1.15
Limestone	0.56
Calcium phosphate dibasic	1.22
Salt	0.78
Sodium bicarbonate	0.45
Premix ^1^	0.89
Total	100.00
Nutrient levels ^2^	
Digestible energy (MJ/kg)	13.36
Metabolizable energy (MJ/kg)	11.14
Dry matter	100.00
Crude protein	17.58
Ether extract	3.86
Ash	11.09
Neutral detergent fiber	45.39
Acid detergent fiber	29.19
Calcium	1.20
Phosphorus	0.44

^1^ The premix provided the following nutrient content for one kilogram of diet: vitamin A, 6000 IU; vitamin D3, 2500 IU; vitamin E, 12.5 IU; vitamin K3, 31.8 mg; vitamin B1, 0.035; vitamin B2, 8.5 mg; vitamin B6, 0.9 mg; nicotinic acid, 22 mg; D-pantothenic acid, 17 mg; vitamin B12, 0.03 mg; biotin, 0.14 mg; folic acid, 1.5 mg; Fe, 0.04 g; Cu, 0.008 g; Zn, 0.05 g; Mn, 0.03 g; I, 0.3 mg; Se, 0.3 mg; Co, 0.25 mg. ^2^ Digestible energy and metabolizable energy was a calculated value, while the others were measured value.

**Table 2:** Nutrient apparent digestibility of mutton sheep fed diets supplemented with different levels of WEAA.

Item ^1^	WEAA Supplemental Level, mg/kg Diet ^2^				SEM	*p*-Value ^3^	
	0	500	1000	1500		Linear	Quadratic
d 1–30							
DM	87.16	86.75	86.34	85.78	0.28	0.122	0.310
CP	74.70	73.44	74.21	73.14	0.38	0.272	0.553
EE	71.38	76.08	74.75	75.65	0.74	0.083	0.084
NDF	43.02	44.64	45.71	44.22	0.54	0.401	0.368
ADF	42.92	42.99	41.26	41.56	0.78	0.488	0.789
Ca	35.52	35.18	34.52	34.02	0.91	0.537	0.828
P	20.01	20.90	19.67	18.90	1.66	0.802	0.946
d 31–60							
DM	86.07	86.67	86.35	85.59	0.37	0.605	0.593
CP	68.78 ^b^	72.41 ^a^	73.28 ^a^	70.62 ^ab^	0.58	0.227	0.008
EE	74.23	75.20	75.04	74.62	0.74	0.878	0.891
NDF	36.88	42.67	43.21	40.57	1.20	0.263	0.127
ADF	36.59	41.83	42.80	40.00	0.98	0.261	0.104
Ca	28.15	30.43	30.18	27.68	1.33	0.798	0.696
P	13.18 ^b^	23.12 ^a^	23.72 ^a^	24.12 ^a^	1.49	0.012	0.030

^1^ DM = Dry matter; CP = Crude protein; EE = Ether extract; NDF = Neutral detergent fiber; ADF = Acid detergent fiber; Ca = Calcium; P = Phosphorus. ^2^ WEAA: Water extracts of *Artemisia annua* L.; Different concentrations of WEAA (0, 500, 1000, and 1500 mg/mL), values were expressed as the means of 8 sheep in each group. ^3^ Values within a row with different superscripts (^a,b^) differ significantly at *p* < 0.05, whereas the probability value of 0.05 ≤ *p* < 0.10 was considered as a tendency.

**Table 3:** Blood biochemical indicators of mutton sheep fed diets supplemented with different levels of WEAA.

Item ^1^	WEAA Supplemental Level, mg/kg Diet ^2^				SEM	*p*-Value ^3^	
	0	500	1000	1500		Linear	Quadratic
d 30							
TP, g/L	67.08 ^b^	71.34 ^ab^	74.42 ^a^	73.61 ^a^	1.02	0.011	0.016
ALB, g/L	27.22 ^b^	31.07 ^ab^	33.46 ^a^	31.07 ^ab^	0.79	0.045	0.014
BUN, mmol/L	7.22	6.82	6.95	6.57	0.13	0.118	0.299
GLU, mmol/L	3.48	3.61	3.75	3.60	0.09	0.519	0.622
TG, mmol/L	0.36	0.32	0.30	0.30	0.02	0.181	0.367
TC, mmol/L	1.37	1.40	1.36	1.39	0.03	0.923	0.995
HDL-C, mmol/L	0.90 ^b^	0.98 ^b^	1.15 ^a^	1.00 ^b^	0.03	0.060	0.024
LDL-C, mmol/L	0.91	0.89	0.84	0.87	0.02	0.520	0.734
CRE, μmol/L	53.40	52.97	51.61	53.10	1.64	0.877	0.956
ALP, U/L	81.61	75.43	77.54	79.33	9.05	0.955	0.976
TBIL, μmol/L	20.76	19.77	18.45	18.37	0.70	0.200	0.430
d 60							
TP, g/L	72.62 ^b^	77.45 ^ab^	81.52 ^a^	79.82 ^ab^	1.23	0.019	0.026
ALB, g/L	27.52 ^b^	33.56 ^a^	35.98 ^a^	33.03 ^ab^	1.04	0.036	0.010
BUN, mmol/L	7.18 ^a^	6.81 ^a^	5.48 ^b^	6.06 ^ab^	0.22	0.020	0.039
GLU, mmol/L	3.11 ^b^	4.28 ^a^	4.77 ^a^	4.36 ^a^	0.18	0.010	0.002
TG, mmol/L	0.22	0.22	0.19	0.18	0.01	0.199	0.436
TC, mmol/L	2.09	2.16	2.10	2.21	0.06	0.857	0.601
HDL-C, mmol/L	1.31	1.32	1.38	1.31	0.02	0.727	0.485
LDL-C, mmol/L	1.17	1.02	0.93	1.03	0.04	0.205	0.105
CRE, μmol/L	56.67	53.75	54.23	55.60	1.45	0.816	0.769
ALP, U/L	65.09	69.55	60.71	68.14	4.20	0.947	0.986
TBIL, μmol/L	18.86	16.33	17.40	18.32	0.84	0.874	0.637

^1^ TP = Total protein; ALB = Albumin; BUN = Blood urea nitrogen; GLU = Glucose; TG = Triglyceride; TC = Total cholesterol; HDL-C = High-density lipoprotein cholesterol; LDL-C = Low-density lipoprotein cholesterol; CRE = Creatinine; ALP = Alkaline phosphatase; TBIL = Total bilirubin. ^2^ WEAA: Water Extracts of *Artemisia annua* L.; Different concentrations of WEAA (0, 500, 1000, and 1500 mg/mL), values were expressed as the means of 8 sheep in each group. ^3^ Values within a row with different superscripts (^a,b^) differ significantly at *p* < 0.05, whereas the probability value of 0.05 ≤ *p* < 0.10 was considered as a tendency.

**Table 4:** Intestinal morphology of mutton sheep fed diets supplemented with different levels of WEAA.

Item ^1^	WEAA Supplemental Level, mg/kg Diet ^2^				SEM	*p*-Value ^3^	
	0	500	1000	1500		Linear	Quadratic
Duodenum							
VH	517.56	540.45	553.82	540.55	9.80	0.356	0.454
CD	158.72 ^a^	151.05 ^ab^	141.68 ^b^	148.06 ^ab^	2.21	0.038	0.029
VH/CD	3.29	3.63	3.82	3.83	0.10	0.049	0.110
Jejunum							
VH	401.80	429.59	471.43	463.53	11.56	0.028	0.066
CD	172.77 ^a^	158.92 ^b^	154.94 ^b^	160.77 ^b^	1.84	0.007	<0.001
VH/CD	2.34 ^b^	2.72 ^ab^	3.05 ^a^	2.89 ^a^	0.091	0.012	0.012
Ileum							
VH	421.83	443.21	463.16	461.94	10.223	0.136	0.292
CD	161.87 ^a^	149.88 ^ab^	143.42 ^b^	151.92 ^ab^	2.239	0.049	0.009
VH/CD	2.597 ^b^	2.988 ^ab^	3.235 ^a^	3.044 ^a^	0.075	0.013	0.006

^1^ VH = Villus height; CD = Crypt depth; VH/CD = Ratio of villus height to crypt depth. ^2^ WEAA: Water extracts of *Artemisia annua* L.; Different concentrations of WEAA (0, 500, 1000, and 1500 mg/mL), values were expressed as the means of 8 sheep in each group. ^3^ Values within a row with different superscripts (^a,b^) differ significantly at *p* < 0.05, whereas the probability value of 0.05 ≤ *p* < 0.10 was considered as a tendency.

**Table 5:** Intestinal digestive enzymes activity of mutton sheep fed diets supplemented with different levels of WEAA.

Item	WEAA Supplemental Level, mg/kg Diet ^1^				SEM	*p*-Value ^2^	
	0	500	100	1500		Linear	Quadratic
α-amylase, U/mgprot							
Duodenum	12.71 ^b^	17.03 ^a^	18.52 ^a^	17.66 ^a^	0.45	<0.001	<0.001
Jejunum	16.69	16.80	15.65	17.30	0.44	0.985	0.726
Ileum	8.66 ^b^	9.95 ^b^	12.08 ^a^	9.55 ^b^	0.39	0.214	0.018
Lipase, U/gprot							
Duodenum	633.08	680.50	654.30	656.46	12.58	0.703	0.633
Jejunum	710.21 ^b^	768.17 ^a^	766.58 ^a^	757.24 ^ab^	7.65	0.133	0.043
Ileum	1068.47	967.95	1082.02	1106.72	41.03	0.547	0.671
Chymotrypsin, U/mgprot							
Duodenum	10.14	11.70	13.42	11.68	0.55	0.218	0.161
Jejunum	10.72 ^b^	19.84 ^a^	20.30 ^a^	17.70 ^a^	1.12	0.005	0.003
Ileum	12.90 ^b^	17.44 ^a^	20.40 ^a^	18.78 ^a^	0.86	0.015	0.004
Trypsin, U/mgprot							
Duodenum	206.29	206.62	241.73	255.27	8.74	0.042	0.124
Jejunum	203.23	207.53	200.19	200.48	7.90	0.835	0.971
Ileum	391.56	388.04	415.49	384.19	7.44	0.683	0.918

^1^ WEAA: Water Extracts of *Artemisia annua* L.; Different concentrations of WEAA (0, 500, 1000, and 1500 mg/mL), values were expressed as the means of 8 sheep in each group. ^2^ Values within a row with different superscripts (^a,b^) differ significantly at *p* < 0.05, whereas the probability value of 0.05 ≤ *p* < 0.10 was considered as a tendency.

**Table 6:** Bacterial enumeration of intestinal flora in mutton sheep fed diets supplemented with different levels of WEAA (Log CFU/g).

Item	WEAA Supplemental Level, mg/kg Diet ^1^				SEM	*p*-Value ^2^	
	0	500	1000	1500		Linear	Quadratic
d 30							
Total Aerobic Bacteria	6.9	6.83	6.82	6.88	0.043	0.853	0.774
*Escherichia coli*	4.82	4.57	4.61	4.6	0.052	0.172	0.209
Total Anaerobic Bacteria	6.52	6.51	6.68	6.61	0.034	0.147	0.329
*Bifidobacteria*	5	5.21	5.24	5.11	0.045	0.347	0.114
*Lactobacilli*	5.08	5.14	5.21	5.28	0.06	0.225	0.485
d 60							
Total Aerobic Bacteria	6.83	6.85	6.84	6.77	0.043	0.683	0.823
*Escherichia coli*	4.79 ^a^	4.54 ^ab^	4.23 ^b^	4.59 ^ab^	0.07	0.176	0.043
Total Anaerobic Bacteria	6.61	6.63	6.69	6.66	0.014	0.11	0.225
*Bifidobacteria*	5.09 ^b^	5.24 ^b^	5.60 ^a^	5.39 ^ab^	0.061	0.021	0.021
*Lactobacilli*	5.16 ^b^	5.36 ^a^	5.33 ^a^	5.26 ^ab^	0.027	0.299	0.036

^1^ WEAA: Water Extracts of *Artemisia annua* L.; Different concentrations of WEAA (0, 500, 1000, and 1500 mg/mL), values were expressed as the means of 8 sheep in each group. ^2^ Values within a row with different superscripts (^a,b^) differ significantly at *p* < 0.05, whereas the probability value of 0.05 ≤ *p* < 0.10 was considered as a tendency.

## Data Availability

The original contributions presented in this study are included in the article/Supplementary Materials. Further inquiries can be directed to the corresponding authors.

## Supplementary Materials

The following supporting information can be downloaded at: https://www.mdpi.com/article/10.3390/ani16020340/s1. Supplementary Table S1: Compound content of WEAA (DM basis, %); Supplementary Figure S1: Total ion current chromatogram of WEAA metabolites in negative ion mode (NEG); Supplementary Figure S2: Total ion current chromatogram of WEAA metabolites in positive ion mode (POS).
