# Supplementary material for: Effect of Water Extract of Artemisia annua L. on Growth Performance, Blood Biochemical Parameters and Intestinal-Related Indices in Mutton Sheep
Source: Animals (Basel). 2026 Jan 22;16(2):340. doi: 10.3390/ani16020340 (PMC12838144; doi:10.3390/ani16020340)
Supplement: Supplementary file 1 [file animals-16-00340-s001.zip › animals-4094304-supplementary.pdf]

Effect of Water Extract of *Artemisia annua* L. (WEAA) on Growth Performance, Blood Biochemical Parameters and Intestinal-related indices in Mutton Sheep

Gen Gang [1], Ruiheng Gao [1], Manman Tong [1], Shangxiong Zhang [2], Shiwei Guo [1], Xiao Jin [1], Yuanyuan Xing [1], Sumei Yan [1], Yuanqing Xu [1,*], Binlin Shi [1,*]

Supplementary Table S1

Compound contents of WEAA (DM basis,%)

Compounds	contents
Organic acids and derivatives	24.61
Soluble polysaccharide	18.64
Flavonoids	9.80
Prenol lipids	7.75
Organoheterocyclic compounds	7.75
Organooxygen compounds	5.01
Nucleosides, nucleotides, and analogues	5.01
Fatty acyls	4.79
Benzene and substituted derivatives	3.87
Glycerophospholipids	2.28
Coumarins and derivatives	2.05
Cinnamic acids and derivatives	1.82
Phenols	1.60
Others	5.01

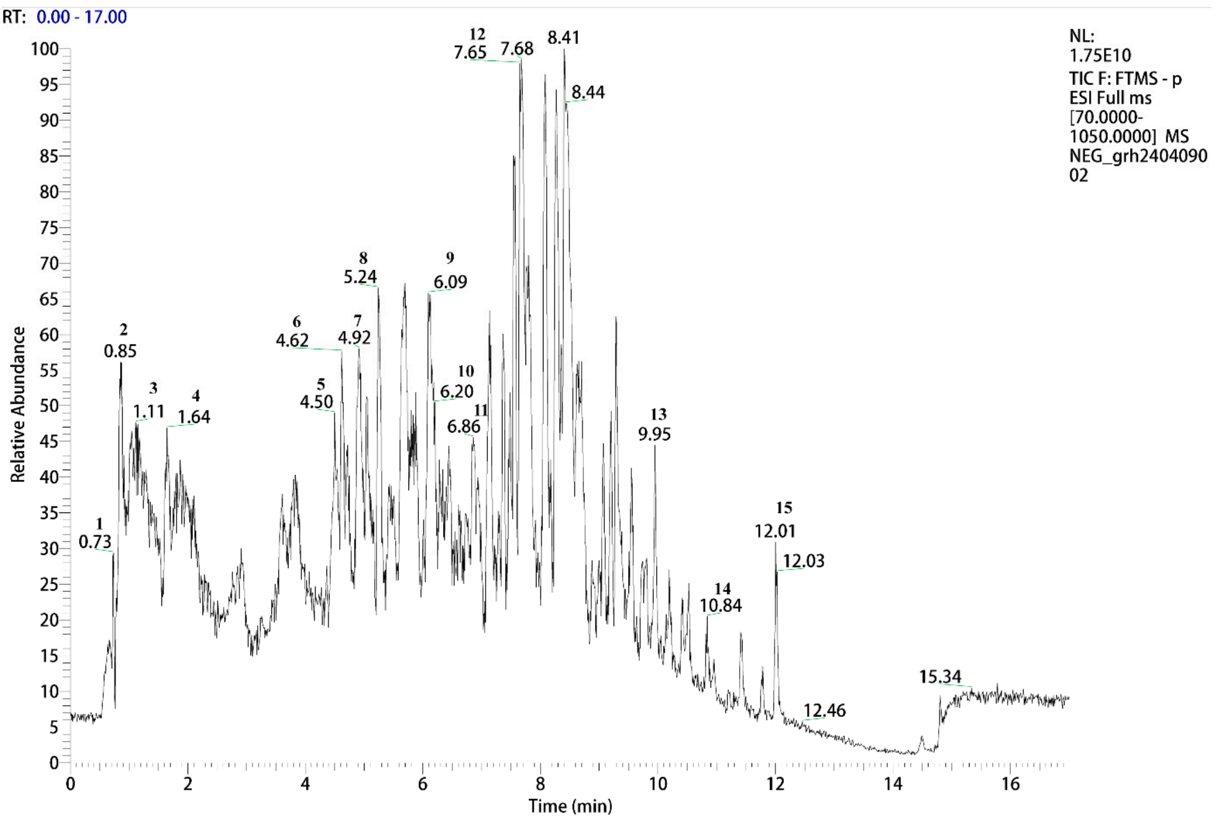

Supplementary Figure S1 Total ion current chromatogram of WEAA metabolites in negative ion mode

(NEG)

Note: 1. Myo-Inositol; 2. QUEBRACHITOL; 3. Unidentified peak; 4. Glucaric acid; 5. 3-O-Caffeoylquinic acid, 1-o-p-Coumaroyl-beta-d-glucose; 6. Cryptochlorogenic acid; 7. Palatinose; 8. Sucrose; 9. Isorhamnetin3-galactoside; 10. p-Hydroxybenzoic acid; 11. Melezitose; 12. (R)-2-Hydroxycaprylic acid; 13. Hydroxyvalerenic Acid; 14. Stearidonic acid; 15. Centaureidin

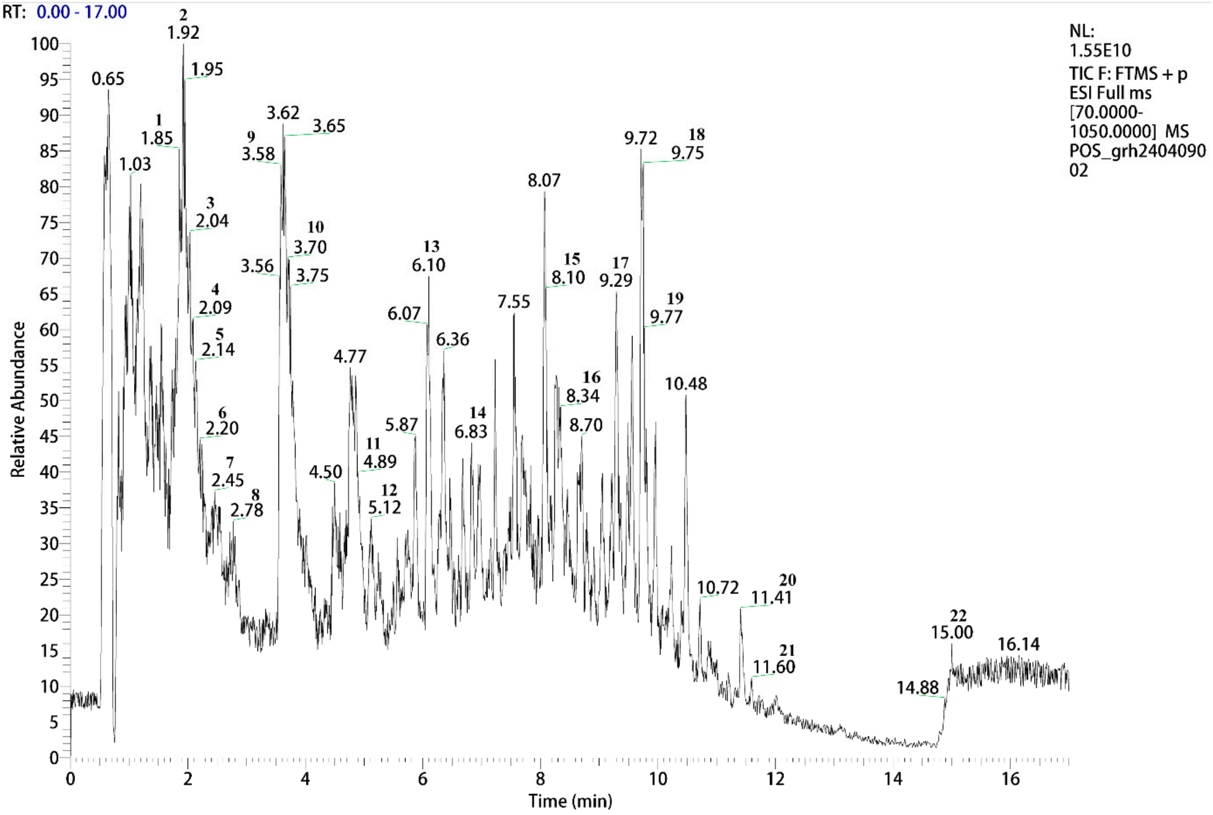

Supplementary Figure S2 Total ion current chromatogram of WEAA metabolites in positive ion mode (POS)

Note: 1. Leucyl-Valine; 2. (E)-Cinnamic acid; 3. Glu Ser Leu, Val Ile Thr, Glu Gln Leu; 4. Ala Ser Ile, Gly Pro Val; 5. Pantothenic acid; 6. 4-hydroxy-3,5-dimethoxybenzoic acid; 7. Glu Thr Ile, Asp Thr Leu; 8. 1-O-(3-Hydroxy-4,5-dimethoxybenzoyl)hexopyranose; 9. Inosine; 10. Val Glu Val; 11. Asp Val Leu; 12. Laminine; 13. Phe Val Phe; 14. 15,16-Dihydrotanshinone I; 15. Aurantio-obtusin, 5,7,3'-Trihydroxy-6,4',5'-trimethoxyflavone, PC(14:0/0:0); 16. N-[(4-methoxyphenyl)methyl]-3-[1-[6-(propylamino)pyrimidin-4-yl]piperidin-4-yl]propenamide; 17. ISOLACTARORUFIN; 18. Arteannuin B; 19. Ilicic acid; 20. Isoalantolactone, 6Beta-Naltrexol; 21. Confertifoline; 22. Casticin
